# In Vitro Release and In Vivo Study of Recombinant TGF-β and EGCG from Dual Self-Cross-Linked Alginate-Di-Aldehyde *In Situ* Injectable Hydrogel for the Repair of a Degenerated Intervertebral Disc in a Rat Tail

**DOI:** 10.3390/gels11080565

**Published:** 2025-07-22

**Authors:** Bushra Begum, Seema Mudhol, Baseera Begum, Syeda Noor Madni, Sharath Honganoor Padmanabha, Vazir Ashfaq Ahmed, N. Vishal Gupta

**Affiliations:** 1 Department of Pharmaceutics, JSS College of Pharmacy, JSS Academy of Higher Education & Research, Sri Shivarathreeshwara Nagar, Mysuru 570015, India; bushrapharma2007@gmail.com (B.B.); sharath.15041993@gmail.com (S.H.P.); 2 Department of Pharmaceutics, Farooqia College of Pharmacy, Mysuru 570021, India; madni2865@gmail.com; 3 Department of Pharmacology, Sardavilas College of Pharmacy, Mysuru, Krishnamurthy Puram, Mysuru 570004, India; seema.mudhol@gmail.com; 4 Department of Clinical Trials, Cheluvamba Hospital, Irwin Road, Mysuru 570001, India; baseera2001@gmail.com; 5 Department of Pharmaceutics, Ikon Pharmacy College, Bidadi 562109, India; mail2vazir@gmail.com

**Keywords:** low back pain, intervertebral disc key, hydrogel, alginate-di-aldehyde, growth factor, epigallocatechin-3-gallate, recombinant-transforming growth factor β, proteoglycan, cellular viability, regeneration

## Abstract

**Background and Objective**: Intervertebral disc degeneration (IVDD) is a leading cause of lower back pain with limited regenerative treatments. Among emerging regenerative approaches, growth factor-based therapies, such as recombinant human transforming growth factor-beta (Rh-TGF-β), have shown potential for disc regeneration but are hindered by rapid degradation and uncontrolled release by direct administration. Additionally, mechanical stress elevates heat shock protein 90 (HSP-90), impairing cell function and extracellular matrix (ECM) production. This study aimed to investigate a dual self-cross-linked alginate di-aldehyde (ADA) hydrogel system for the sustained delivery of Rh-TGF-β and epigallocatechin gallate (EGCG) to enhance protein stability, regulate release, and promote disc regeneration by targeting both regenerative and stress-response pathways. **Methods**: ELISA and UV-Vis spectrophotometry assessed Rh-TGF-β and EGCG release profiles. A rat tail IVDD model was established with an Ilizarov-type external fixator for loading, followed by hydrogel treatment with or without bioactive agents. Disc height, tissue structure, and protein expression were evaluated via radiography, histological staining, immunohistochemistry, and Western blotting. **Results**: The hydrogel demonstrated a biphasic release profile with 100% Rh-TGF-β released over 60 days and complete EGCG release achieved within 15 days. Treated groups showed improved disc height, structural integrity, and proteoglycan retention revealed by histological analysis and elevated HSP-90 expression by immunohistochemistry. In contrast, Western blot analysis confirmed that EGCG effectively downregulated HSP-90 expression, suggesting a reduction in mechanical stress-induced degeneration. **Conclusions**: ADA hydrogel effectively delivers therapeutic agents, offering a promising strategy for IVDD treatment.

## 1. Introduction

The intervertebral disc (IVD), the softest and largest avascular tissue in the human body, made from a fibrocartilage structure, is a unique organ sandwiched between each vertebra [1,2]. Being a weight-bearing organ, its functions are to serve as a shock absorber and improve the strength and fluidity of spinal movements by supporting our heads and heavy loads that safeguard our spinal cord, nerve roots, and provide flexibility to the column. Serving as a ligament to keep the spine’s vertebrae together, the IVD comprises three discrete regions: (a) Nucleus pulposus (NP); (b) Annulus fibrosus (AF); and (c) Cartilage endplate [3]. The NP is a centrally situated gelatinous structure that produces an extracellular matrix (ECM). The NP’s functions include evenly redistributing the forces throughout the spine and resisting compressive pressures. The AF is a thick, dense structure that endures the tension produced during NP deformation, which gives stiffness to the tissue [4,5]. The cartilage endplate (CEP) is made up of hyaline cartilage. The process of the diffusion of nutrients via vascularised vertebrae, where solutes pass into the NP, is supplied by the CEP. However, the unbalanced state of the IVD gets worse with age; additionally, environmental factors such as smoking, obesity, genetics, and biomechanical loads lead to the cause of IVDD (intervertebral disc degeneration) that is associated with lower back pain [6,7]. There are enough viable cells in the early stages of degeneration; fewer viable cells actively appear in the mid stages, and the ultimate level of degeneration is reached in the severe stage. The process of regenerative tissue repair, in which damaged tissue is entirely repaired to its pre-injury condition through new growth via mechanisms that permit the transformation of cells into matrices, ought to be included. One could categorize this as tissue engineering. Hence, biological repair with growth factor (GF) therapy may be required to regenerate the degenerative IVD as a minimally invasive therapy to start and stimulate the early stages of cellular remodelling residing in IVDs. These GFs are polypeptides that increase the matrix metabolism by stimulating cell proliferation, differentiation, migration, and ECM synthesis (PG synthesis, collagen synthesis) within the degenerated disc [8]. Transforming growth factor-β (TGF-β) is a member of the anabolic GF belonging to the superfamily. It is found in many different tissues, including the bone matrix, platelets, activated lymphocytes, and cartilage. The three TGF-β isotypes, known as β1, β2, and β3, are homologous homodimers. They serve as structurally related homo- or heterodimers connected by a single disulfide link. More than 35 members comprise the TGF-β family, including TGF-βs, activins, and bone morphogenetic proteins (BMPs). Recent in vitro and in vivo studies have demonstrated that the use of TGF-β enhances the synthesis of collagen, fibronectin, and proteoglycans—the components of the extracellular matrix—by promoting chondrocyte production, facilitating cell migration and differentiation, inducing apoptosis, and inhibiting matrix breakdown, to regulate disc cell metabolism in order to repair disc degeneration; as a result, they are important in the healing processes that follow cartilage damage. Another significant anti-inflammatory characteristic of TGF-β is that it prevents monocytes and macrophages from releasing inflammatory cytokines [9,10,11]. The drawbacks of the direct injection of GFs are a short half-life, protein instability, and enzymes at body temperature, which quickly deactivate their particular properties, as well as their incorrect localization in the target area [12,13,14]. Direct loading of GFs into a polymeric matrix, like a hydrogel, can increase the biological stability of GFs in tissue engineering. During the initial swelling phase, typical release profiles show a rapid burst release followed by a prolonged release of a particular amount of protein that was held back by the gel network [15,16]. As a scaffold material, hydrogels are three-dimensional networks of cross-linked polymers that replicate the mechanical and biological characteristics of the NP. They can swell in water and retain a large amount of water without losing their structure because of their chemically or physically cross-linked individual polymer chains. Thus, it functions as a synthetic ECM due to its soft, flexible, biocompatible nature, adjustable degradability, and adequate permeability to nutrients and oxygen. The ability of hydrogels to retain bioactive substances like drugs and GFs is an additional advantage [17,18,19,20]. The range of proteins that cells produce in response to external stimuli such as heat, mechanical force, or chemical poisons is referred to as “stress proteins” or “heat shock proteins (HSPs)” [21]. These are present in the cytoplasm and various intracellular spaces. These HSPs play a role in many cellular functions, such as cell protection, proliferation, survival, differentiation, immunological response, signal transduction pathway regulation, neuroprotection, and cell death. As a result, they hep prevent cartilage damage. In IVD, they it was shown that HSP90 overexpression was up-regulated following static compression (load) [22,23]. On the other hand, overexpression of HSP-90 reverses the activity in response to extended stress or load, which inhibits cellular protein, stops cell development or differentiation, and results in apoptosis or pro-death effects on cells [24]. Epigallocatechin-3-gallate (EGCG) is the primary polyphenol which is isolated from Camellia sinensis green tea. Over the last ten years, the biological potential of EGCG has been widely investigated, demonstrating its ability to suppress inflammatory responses in IVD cells and function as an HSP-90 inhibitor to reduce the overexpression of HSP-90 [25]. This study aims to evaluate the therapeutic potential of a dual self-cross-linked alginate di-aldehyde (ADA) hydrogel as a biodegradable delivery system for recombinant TGF-β and EGCG in the treatment of IVDD, wherein the hydrogel is designed to degrade gradually and release the encapsulated bioactive molecules in a controlled manner. The research evaluates the effects of these bioactive molecules on disc height, structural integrity, and proteoglycan retention through histological analyses, including H&E and Safranin-O staining. Immunohistochemistry (IHC) analysis showed a considerable rise in HSP-90 expression in degenerated IVDs. Western blot (WB) analysis was used to evaluate HSP90 expression using EGCG (50 µm) and its role in IVDD pathophysiology. The study highlights EGCG’s ability to downregulate HSP90, enhance ECM production, and support chondrocyte viability of the GF under mechanical stress. Findings suggest that the developed hydrogel formulation is injectable, biodegradable, and provides a supportive microenvironment for the delivery of bioactive molecules, particularly Rh-TGF-β (500 ng) and EGCG (50 µm), which may improve disc regeneration, maintaining cell viability, proteoglycan levels, and counteracting degenerative changes, offering a promising therapeutic strategy for IVDD treatment.

## 2. Results and Discussion

### 2.1. Oxidation of Sodium Alginate and UV–Vis-Based Determination of Degree of Oxidation

Sodium alginate was oxidized using freshly prepared sodium metaperiodate in the dark, yielding alginate di-aldehyde (ADA) with 54% and 70% degrees of oxidation. ADA with 70% oxidation showed greater structural modification due to increased cleavage at the C-2 and C-3 positions and more aldehyde group formation. This led to ring opening and backbone disruption, producing a flexible polymer suitable for crosslinking and drug delivery. Higher oxidation (70%) also enhanced biodegradability and gelability with 9% gelatin, making it more applicable for biomedical use. The detailed methodology and characterization data supporting these findings are provided in *Bushra Begum et al*. [26].

### 2.2. Gelation Profile and Rheological Evaluation of the Hydrogel Formulation

An increased degree of oxidation significantly enhanced gelability in the presence of 9% gelatin, improving the hydrogel’s suitability for biomedical applications. Gelation behaviour was influenced by the oxidation level, with 70% oxidized ADA forming more stable and faster-setting hydrogels than the 54% variant. The dual self-crosslinked hydrogel comprising 7% ADA (70%) + 0.1 M borax + 9% gelatin + 1% PEG exhibited gelation within 2.5 min at 37 °C. Rheological analysis showed G′ surpassing G′′ at 2.5 min, indicating gelation. Including 1 min of mixing, total gelation time was approximately 3.5 min, aligning with experimental observations which closely corresponds with the experimentally observed gelling time. Comprehensive details of the methodology and characterization supporting these findings are available in *Bushra Begum et al*. [26].

### 2.3. In Vitro Degradation Behaviour of the Hydrogel

The 7% ADA (70%) + 0.1 M borax + 9% gelatin + 1% PEG hydrogel showed ~80% degradation at pH 6.5 over 60 days. Dual crosslinking enhanced stability. The system enabled the sustained release of bioactive agents, supporting its potential for long-term therapeutic delivery applications.

### 2.4. In Vitro Release of (Rh-TGF-β) Recombinant Transforming Growth Factor-Beta and Epigallocatechin-3-Gallate (EGCG)

**Figure 1:**
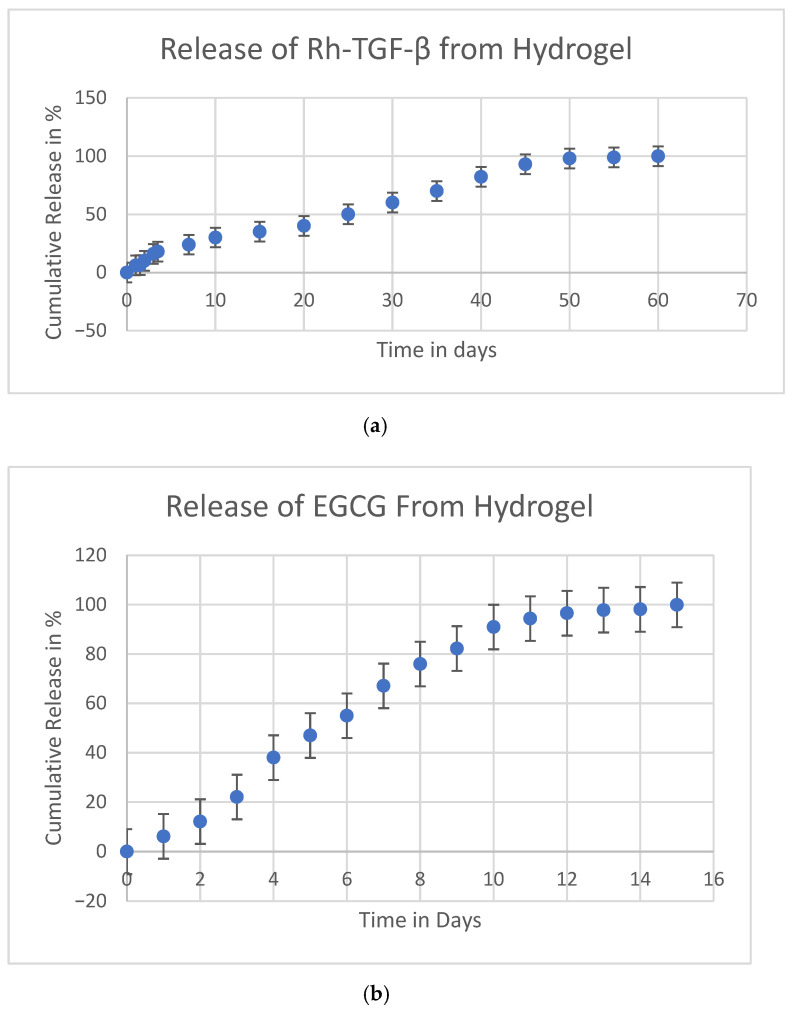
The cumulative release of (**a**) Rh-TGF-β (n = 3) Standard deviation found to be 0.1 to 0.4509 and (**b**) EGCG loaded from dual self-cross-linked ADA in situ injectable hydrogel (n = 3) Standard deviation found to be 0.1 to 0.5507.

One of the key challenges in GF therapy is the instability of proteins, as GFs possess a short half-life and are susceptible to rapid degradation due to enzymatic activity at body temperature when delivered through direct administration. Additionally, they often lack precise localization in the target area, leading to inefficient therapeutic effects. These limitations reduce their efficacy in tissue regeneration and necessitate an optimized delivery system to enhance their stability and controlled release. Hydrogel-based delivery systems have been explored to address these issues. By incorporating GFs into hydrogel matrices, their loss during tissue regeneration can be minimized, and their bioactivity can be maintained for a prolonged duration. The dual self-cross-linked alginate di-aldehyde hydrogel has been identified as an effective vehicle for the release of recombinant transforming growth factor-beta (Rh-TGF-β). As illustrated in Figure 1a, the release profile of Rh-TGF-β from the dual self-cross-linked alginate di-aldehyde hydrogel exhibits a characteristic biphasic pattern. Initially, there is a burst release phase, where a large dose of Rh-TGF-β is rapidly released, ensuring an immediate therapeutic effect. This phase is crucial as it provides the patient with a high concentration of the GF during the early stages of treatment. Following the initial burst, the hydrogel enables a gradual and controlled release of Rh-TGF-β over a period of 60 days up to 100% at pH 6.5, which corresponds to the pH of a degenerated disc. The hydrogel’s high swelling capacity, porous structure, and controlled degradation enable a sustained and extended diffusion of encapsulated bioactive molecules. Therefore, the release mechanism is a combination of diffusion and degradation-driven erosion, which not only facilitates prolonged release but also protects Rh-TGF-β from enzymatic degradation and ensures its localized retention at the injury site. The dual self-cross-linked alginate dialdehyde hydrogel is capable of stabilizing a number of GFs, preventing premature degradation, and ensuring their localized and sustained bioactivity at the target site. By maintaining a controlled release pattern, this hydrogel-based system enhances the efficiency of Rh-TGF-β in promoting tissue regeneration and repair. Thus, the hydrogel facilitates the stabilization of Rh-TGF-β, maintaining its structural integrity and biological function. This controlled environment prevents the loss of Rh-TGF-β, ensuring that the GF remains bioavailable at the degenerated site for several days, thereby enhancing its therapeutic potential.

The cumulative release kinetics of EGCG, another bioactive molecule encapsulated within the hydrogel, was evaluated over a 15-day period. As shown in Figure 1b, up to 100% (plateau) of EGCG was released within 15 days. This controlled release was significantly influenced by the porous structure and degradation properties of the hydrogel, which contains aldehyde functional groups that enhance its degradability and regulate the diffusion of encapsulated molecules. At the same time, it facilitates an efficient release mechanism. This property is particularly beneficial in tissue engineering applications, where a gradual and continuous supply of bioactive molecules is essential for promoting cell proliferation, matrix remodelling, and overall tissue repair.

### 2.5. Radiological Results

**Figure 2:**
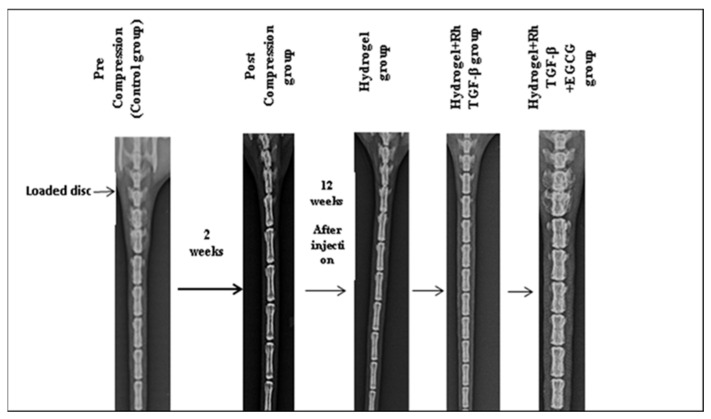
Lateral radiographs of rat caudal spine pre-compression and post-compression groups, 2 weeks after load and 12 weeks after treatment with injection.

**Figure 3:**
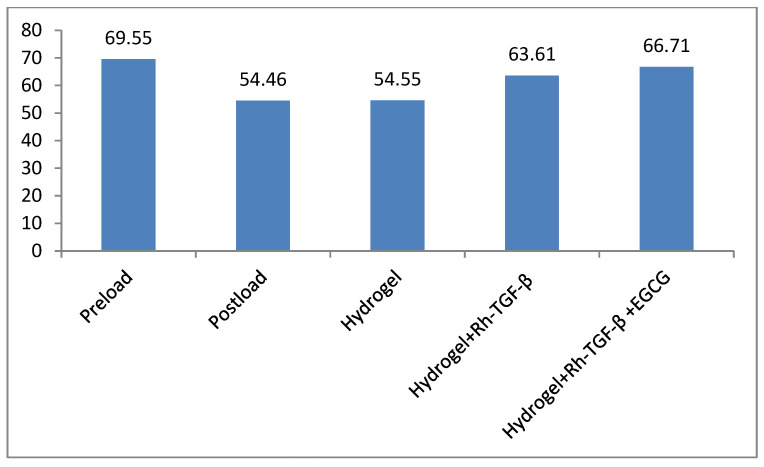
As per the digital X-ray, the disc height Preload and Postload (n = 3) and Hydrogel, Hydrogel + Rh-TGF-β and Hydrogel + Rh-TGF-β + EGCG (n = 6).

During the study period, the animals either maintained or slightly increased their body weight and exhibited normal activity levels and behaviours, indicating that they tolerated the loading operations well. Mild inflammation was observed around the pins in certain rats, which was managed effectively using a topical application of anti-inflammatory drugs. IVD height was measured and calculated by averaging multiple measurements, with numerical units used for consistency. As illustrated in Figure 2 and Figure 3, the control group had an initial disc height of 69.55 U before loading. However, after the loading phase, the disc height significantly decreased, reaching the lowest value of 54.46 U in the post-loading phase. Following the injection of different treatments, variations in disc height were observed. The hydrogel-treated group exhibited a disc height of 54.55 U, indicating a minimal improvement. In contrast, the Hydrogel + Recombinant TGF-β group demonstrated a substantial increase in disc height, reaching 63.61 U, suggesting the beneficial effects of recombinant TGF-β in promoting disc regeneration. Furthermore, the Hydrogel + Recombinant TGF-β + EGCG group exhibited the highest disc height of 66.71 U, indicating an enhanced regenerative effect when the HSP-90 inhibitor was incorporated into the treatment. These findings suggest that changes in disc height correlate with the release of recombinant TGF-β (Rh-TGF-β) and EGCG from the hydrogel. The results highlight the potential of hydrogel-based delivery systems in facilitating the release of bioactive molecules, which may contribute to IVD repair and regeneration. Statistical analysis revealed that the differences in disc height among the groups were statistically significant (*p* < 0.05), thereby underscoring the effectiveness of the applied treatment strategies.

### 2.6. Histologic Results

**Figure 4:**
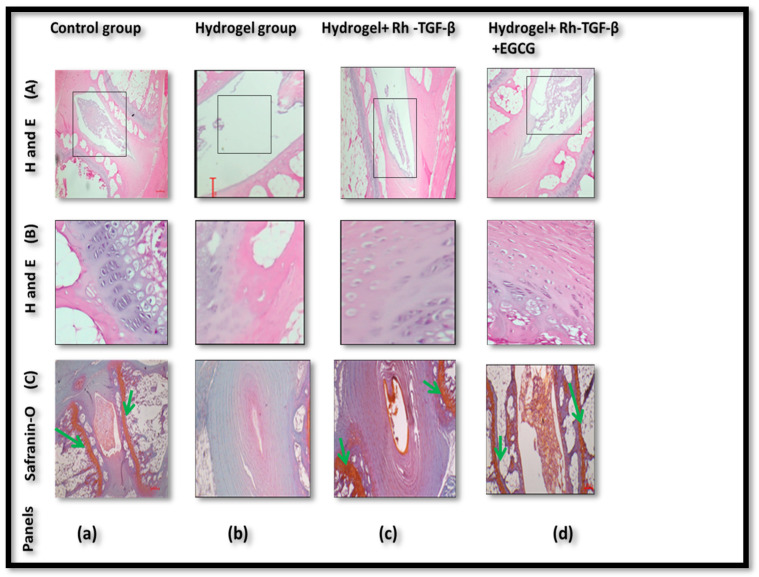
Representative H&E, S-O staining of different groups of disc samples was obtained at 12 weeks for each group after injection. (**A**)—(panel (**a**)) showed that the control group NP tissue was uniformly distributed and well-organized. (Panel (**b**)) NP tissue disappeared or was destroyed. (Panel (**c**)) the hydrogel + Rh-TGF-β group showed that NP tissue was improved, and (panel (**d**)) the hydrogel + Rh-TGF-β + EGCG group showed a distinct dispersion of the NP. (**B**) Produced cellularity (indicates there are more chondrocyte-like cells at the NP–EP junction) in the treated group (panel (**c**)) and (panel (**d**)), which was better than that of (panel (**b**)) and comparable to the control group (panel (**a**)). (**C**) Representative S-O staining indicates more proteoglycan retention and staining intensity in (panel (**c**)) and (panel (**d**)).

In Figure 4A (control group panel a), the IVD is visible with a well-organized NP and surrounding AF. The rectangular boxed area highlights the intact disc structure, including preserved NP architecture and clear demarcation from the AF. In Figure 4A (hydrogel group panel b), the boxed area shows the IVD region where substantial tissue loss is evident. The NP appears degraded or absent, reflecting advanced disc degeneration. In Figure 4A (Hydrogel + Rh-TGF-β group panel c), the disc structure is relatively preserved compared to the hydrogel-only group. The boxed region identifies the area of the NP and AF with improved architecture, indicating regeneration. In Figure 4A (Hydrogel + Rh-TGF-β + EGCG group panel d), the boxed area of the IVD reveals the most intact and cellular NP and AF region among the treated groups, suggesting a synergistic therapeutic effect. In Figure 4B (control group, panel a), the NP region displays a well-organized cellular arrangement with uniform matrix distribution. The boundary between the NP and AF is distinct, and no signs of structural disruption are evident. This serves as a baseline for normal disc morphology. In Figure 4B (Hydrogel group, panel b), the NP region exhibits clear signs of degeneration. There is a noticeable reduction in cell density and disorganization of the ECM structure. The tissue appears fragmented, and the NP–AF boundary is poorly defined, indicating that hydrogel alone offers limited regenerative support. In Figure 4B (Hydrogel + Rh-TGF-β group, panel c), improved tissue restoration is observed. The NP region contains more cells compared to the hydrogel-only group, and the ECM appears denser. However, the tissue is not fully restored to control levels, suggesting that while Rh-TGF-β supports regeneration, in Figure 4B (Hydrogel + Rh-TGF-β + EGCG group, panel d), the NP shows the highest level of cellularity and structural organization among the treatment groups. Migrating cells from the endplate, arranged linearly and directed toward the NP and inner AF, are clearly visible. These cells have a spherical morphology and single nucleus, indicating active cellular involvement in tissue regeneration. The ECM is densely packed and well-preserved, highlighting the enhanced reparative potential of the combined Rh-TGF-β and EGCG therapy. These observations from H&E staining (Figure 4B) support the hypothesis that dual delivery promotes not only cell migration but also improved ECM integrity, contributing to disc repair. In addition, Figure 4C (Safranin-O staining) provides insights into proteoglycan content, an essential component of NP health. In Figure 4C (control group panel a), the NP region stains intensely red (highlighted by green arrows), indicating abundant proteoglycans in a healthy disc. In Figure 4C (hydrogel group panel b), staining is faint or absent in the NP region, pointing to a severe loss of proteoglycans. In Figure 4C (Hydrogel + Rh-TGF-β group panel c), Safranin-O positivity shows the preservation of proteoglycans (highlighted by green arrows). In Figure 4C (Hydrogel + Rh-TGF-β + EGCG group panel d), intense red staining (highlighted by green arrows) indicates robust proteoglycan retention in the NP matrix, supporting effective ECM maintenance. Collectively, these histological findings across Figure 4A–C demonstrate that while hydrogel alone provides no structural support, the incorporation of Rh-TGF-β improves tissue regeneration, and the combination of Rh-TGF-β and EGCG yields the most significant enhancement in cellularity, ECM organization, and proteoglycan retention—hallmarks of effective IVD regeneration.

### 2.7. Immunohistochemistry Results

**Figure 5:**
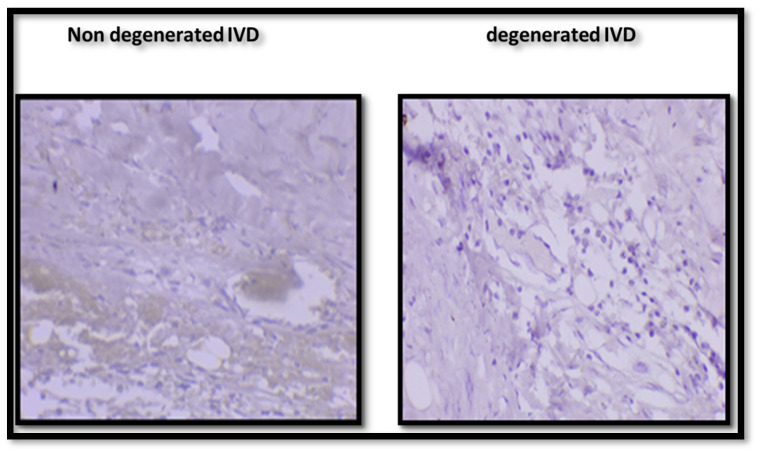
Immunohistochemistry staining of HSP90 in non-degenerated and degenerated rat IVDs.

We initially examined the expression of HSP-90 in rat IVD tissues to investigate its potential role in load-induced IVD degeneration. IHC analysis revealed a significant increase in HSP-90 expression in degenerated IVDs as shown in Figure 5, suggesting that HSP-90 may play a critical role in the pathophysiology by reducing the chondrocyte cells and inhibiting ECM production, leading to further damage to the IVD. Based on these findings, we selected a 50 µM concentration of EGCG for subsequent experiments. While the GF alone resulted in cells and ECM production, the addition of EGCG significantly enhanced its effect, demonstrating its potential therapeutic benefit. To further assess the effect of EGCG treatment on HSP-90 expression, a WB analysis was conducted. The results demonstrated that treatment with 50 µM EGCG effectively inhibited HSP-90 expression in disc tissues subjected to compressive loading. This suggests that EGCG has a protective effect against load-induced IVDD by downregulating HSP-90, potentially contributing to disc regeneration and improved cellular homeostasis.

### 2.8. Western Blotting Results

**Figure 6:**
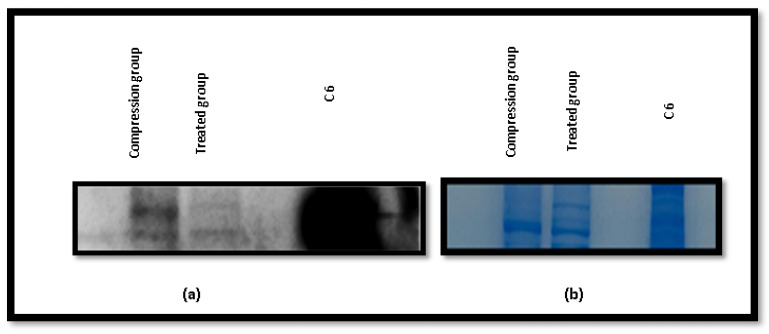
(**a**) WB analysis of HSP90 in the compression group and treated group with C6 rat glioma cell line as a positive control, (**b**) 10% SDS PAGE gel stained with Coomassie Blue to ensure the equal loading of proteins.

Western blot (WB) analysis was performed to investigate the expression of Heat Shock Protein 90 (HSP90) in the compression group and the treated group (hydrogel + TGF-β + EGCG). Immunoblots were developed using the enhanced chemiluminescence (ECL) detection system (BIO-RAD Clarity™ Western ECL substrate from Bio-Rad Laboratories, Hercules, CA, USA), and protein bands were visualized using a gel documentation system (UVI-TEC Alliance 9 system, UVItec Limited, Cambridge, UK). A distinct band corresponding to HSP90 was observed in both the compression group and treated group, confirming the successful detection of the protein, as shown in Figure 6a. However, the absence of bands in the compression group, despite testing multiple options, indicated a potential issue with the detection of these compression groups. To ensure that equal amounts of protein were loaded into each lane and that efficient transfer of proteins from the gel to the polyvinylidene fluoride (PVDF) membrane occurred, the gel was stained with Coomassie blue post-transfer as shown in Figure 6b. The uniform staining confirmed consistent sample loading and efficient protein transfer. The results of the WB analysis demonstrated that treatment with 50 µM EGCG inhibited the overexpression of HSP90 in disc samples subjected to compression. This finding suggests a potential regulatory effect of EGCG on HSP90 expression under mechanical stress conditions, providing insights into its role in cellular stress response mechanisms.

### 2.9. Discussion

IVDD is a progressive condition that affects spinal function and is a leading cause of chronic back pain [27]. The present study explored the potential of a dual self-cross-linked in situ injectable biodegradable hydrogel as a delivery system for Rh-TGF-β and EGCG to address IVDD. The TGF-β family comprises more than 35 members, including TGF-βs, activins, and bone morphogenetic proteins (BMPs). These proteins are essential for tissue development and homeostasis, regulating key cellular processes such as proliferation, differentiation, apoptosis, and migration, in addition to overseeing ECM synthesis [9]. The findings highlight the effectiveness of this system in stabilizing GFs, promoting disc regeneration, and counteracting stress-induced degeneration. One of the major challenges in regenerative medicine is the instability and rapid degradation of GFs following direct administration [15]. The hydrogel-based system used in this study addresses these limitations by providing a controlled release mechanism [28]. The biphasic release pattern of Rh-TGF-β from the dual self-cross-linked alginate di-aldehyde hydrogel, characterized by an initial burst followed by a sustained release over 60 days with 100%, EGCG, a bioactive polyphenol naturally occurring compound found in green tea, potential as a neuroprotective agent, inhibits inflammatory responses in IVD cells, protects NPCs from apoptosis and ECM degradation under oxidative stress through a Bmal1-dependent mechanism and is also known for its anti-inflammatory and antioxidant properties, has shown promise in inhibiting HSP-90 overexpression, which is implicated in IVDD progression [25,29]. The release profile of EGCG from the hydrogel over 15 days, with up to 100% release, further supports it’s potential to provide continuous therapeutic benefits. It ensures both immediate and long-term therapeutic effects. The burst phase delivers a high concentration of GFs to the degenerated disc, while the controlled release phase maintains its bioavailability for prolonged periods, which is crucial for effective tissue regeneration [30]. The covalent dual crosslinking also prolongs hydrogel degradation due to a higher degree of oxidation and lower molecular weight compared to native alginates while allowing swelling and gradual degradation at pH 6.5 over a period of 60 days, as demonstrated in a previous in vitro study (*Bushra Begum et al.*) [26]. While the current study focuses primarily on in vivo regenerative outcomes, the in vivo reabsorption of the hydrogel + Rh-TGF-β + EGCG is supported by histological and structural improvements observed in the treated group over time, indirectly suggesting material resorption and tissue integration. Moreover, the hydrogel prevents the premature degradation of Rh-TGF-β and EGCG, maintaining their structural integrity and biological activity. The ability of this system to localize GFs at the target site minimizes systemic side effects and enhances the therapeutic impact. As a result, hydrogel-based delivery appears to be a promising approach for IVDD treatment. Further disc height measurements provided quantitative evidence of the regenerative potential of hydrogel-based treatments. The control group exhibited an initial disc height of 69.55 U before loading, which significantly decreased to 54.46 U post-loading, reflecting disc degeneration. Following treatment interventions, disc height varied among groups. The hydrogel-only group showed a slight increase to 54.55 U, while the hydrogel + recombinant TGF-β group demonstrated a more substantial recovery to 63.61 U, indicating the regenerative potential of recombinant TGF-β. Notably, the Hydrogel + Recombinant TGF-β + HSP-90 Inhibitor group achieved the highest disc height of 66.71 U. Importantly, statistical analysis confirmed that the differences in disc height among the groups are statistically significant (*p* < 0.05), highlighting the efficacy of the treatment strategies. Suggesting a synergistic effect of the combined therapy. These trends suggest that the restoration of disc height may be linked to the controlled release of Rh-TGF-β and EGCG from the hydrogel system. This suggests that inhibiting HSP-90, a molecular chaperone linked to stress-induced disc degeneration, further enhances regenerative outcomes. These results highlight the potential synergistic effects of GF therapy and HSP-90 inhibition in restoring disc height and function.

Haematoxylin and Eosin (H&E) staining provided further insights into tissue integrity across different experimental groups. The control group maintained a well-organized NP structure, whereas the hydrogel-alone group exhibited significant tissue degeneration, indicating that hydrogel alone does not provide sufficient protection against IVDD. However, the Hydrogel + Rh-TGF-β group showed substantial structural improvement, with preserved tissue architecture. The most significant improvements were observed in the hydrogel + Rh-TGF-β + EGCG group, where the NP exhibited greater structural organization and cellular integrity, indicating the improved effectiveness of Rh-TGF-β in combination with EGCG, which promoted the tissue organization and cellular viability. Safranin-O (S-O) staining further supported these findings by demonstrating enhanced proteoglycan retention in treated groups. The loss of proteoglycans in the hydrogel-alone group suggests that the hydrogel itself does not prevent matrix degradation. However, in the hydrogel + Rh-TGF-β + EGCG group, proteoglycan content was significantly preserved, as indicated by higher staining intensity. This suggests that overexpression of HSP-90 inhibition plays a role in maintaining ECM integrity, which is essential for disc function. HSP-90 is a stress-related protein that has been implicated in IVDD. IHC analysis revealed increased HSP-90 expression in degenerated discs, suggesting that it plays a key role in IVDD pathophysiology. Increased levels of HSP-90 are known to promote chondrocyte apoptosis and inhibit ECM production, leading to progressive disc damage. To counteract the detrimental effects of HSP-90, the study investigated the use of EGCG, a natural polyphenol with known HSP-90 inhibitors and anti-inflammatory properties. WB analysis demonstrated that treatment with 50 µM EGCG effectively downregulated HSP-90 expression in discs subjected to compressive loading. Furthermore, while Rh-TGF-β alone has an effect on cellular activity and ECM production, its efficacy is significantly enhanced when delivered via hydrogel. A single dose of 500 ng Rh-TGF-β administered through a hydrogel system resulted in marked disc regeneration at 12 weeks. In contrast, a 200 ng dose of Rh-TGF-β without hydrogel required multiple injections over a six-week period to produce only a modest stimulatory effect on annular fibro chondrocytes [31]. These findings suggest that hydrogel-based delivery of Rh-TGF-β substantially improves its regenerative potential compared to repeated injections without a delivery matrix. An in vitro study investigated IVD cells in one of the works and has shown that there is no significant toxicity of EGCG at concentrations of 5, 10, and 20 µM [32]. In total, 40 µM of EGCG showed the best therapeutic effect, which protects NPCs (NP) against apoptosis and ECM degradation under oxidative stress in a Bmal1-dependent manner [33]. In comparison, the 50 μm concentration demonstrated an intermediate yet enhanced therapeutic effect, indicating its potential as a balanced and effective dose for further investigation. This suggests that EGCG has a protective effect against mechanical stress-induced IVDD by reducing HSP-90 activity. Thus, Rh-TGF-β, in combination with EGCG, significantly enhanced cellular proliferation and matrix synthesis. This indicates that EGCG not only inhibits degeneration but also potentiates the regenerative effects of Rh-TGF-β. These findings provide strong evidence for the role of HSP-90 inhibition as a novel therapeutic strategy for IVDD treatment.

## 3. Conclusions

The study demonstrated the potential of a dual cross-linked *in situ* injectable biodegradable hydrogel as an effective delivery system for recombinant human Rh-TGF-β and EGCG in addressing IVDD. The controlled biphasic release of Rh-TGF-β ensured both immediate and controlled therapeutic effects, while EGCG contributed to reducing HSP-90 overexpression, thereby preventing stress-induced degeneration. The hydrogel-based system successfully enhanced disc height restoration, preserved ECM integrity, and promoted cellular viability, indicating its potential for IVDD treatment. Histological analysis further confirmed the synergistic effectiveness of Rh-TGF-β with EGCG in improving tissue organization and proteoglycan retention. Additionally, WB analysis validated EGCG’s role in downregulating HSP-90 expression, preventing further disc damage. These findings highlight the promise of hydrogel-based GF and HSP-90 inhibition therapy as a novel approach for IVDD regeneration and repair.

## 4. Materials and Methods

### 4.1. Materials

Recombinant TGF-β GFs 97% purity were purchased from Juniper Life Sciences, Hyderabad, India. EGCG (98% purity) were purchased from TCI America, Tokyo Chemical Industry Co., Ltd., Portland, OR, USA. The ELISA kit was obtained from Elabscience, Inc., New Delhi, India. Haematoxylin were purchased from Loba Chemie Pvt. Ltd., Mumbai, Maharashtra, India. Eosin Y disodium salt were purchased from HiMedia Laboratories Pvt. Ltd. Maharashtra, India, EDTA-(CAS RN: 60-00-4) were purchased from TCI Chemicals (India) Pvt. Ltd., Chennai, Tamil Nadu, India, formaldehyde were purchased from Sigma-Aldrich, Merck Specialities Pvt. Ltd., Mumbai, Maharashtra, India, paraffin—were purchased from Merck Specialities Pvt. Ltd., Mumbai, Maharashtra, India. Tris-EDTA were purchased from HiMedia Laboratories Pvt. Ltd., Thane (W) Maharashtra, India. Bovine serum albuminwere purchased from Otto Chemie Pvt. Ltd., Mumbai, Maharashtra, India. DAB (3,3-Diaminobenzidine) were purchased from Thermo Fisher Scientific Pvt. Ltd., Mumbai, Maharashtra, India. BCA Protein Assay Kit were purchased from Thermo Fisher Scientific, Rockford, IL, USA. PVDF membrane were purchased from Invitrogen, Thermo Fisher Scientific, Carlsbad, CA, USA), HSP90α primary antibody (ABclonal, Cat no-A5006) were purchased from ABclonal Technology, Wuhan, China.

### 4.2. Animal Care and Use

Twenty-seven healthy Wistar rats (180–220 g, 3–4 months old) were obtained from the Laboratory Animal Centre. The study adhered to the guidelines for laboratory animal care and was approved by the Institutional Animal Ethics Committee (IAEC) of KCC Labs, Tumakuru, Karnataka, India, having the proposal number KCC/IAEC/048/2023. Throughout the experiment, the animals had unrestricted access to water and a standard diet, and the guidelines instructed by IAEC throughout the experiment were followed.

### 4.3. Preparation and Determination of Alginate-Di-Aldehyde (ADA)

To prepare alginate di-aldehyde (ADA) with varying degrees of oxidation, 20 g of sodium alginate was initially suspended in 100 mL of ethanol to ensure uniform dispersion and prevent premature dissolution. In a separate beaker, different amounts of sodium metaperiodate were dissolved in 100 mL of deionized (DI) water, and this solution was slowly added to the alginate suspension to obtain alginate samples with different oxidation levels. The reaction mixture was stirred magnetically in the dark for 6 h to prevent light-induced degradation of periodate. After the reaction, the mixture was dialyzed using a dialysis membrane (MWCO ~ 14,000 Da) against 2.5 L of distilled water for 48 h, with regular water changes to remove unreacted periodate and by-products. The dialyzed solution was then lyophilized to obtain dry ADA, which was stored in a desiccator to prevent moisture absorption. The degree of oxidation was subsequently determined by UV–Visible spectroscopy using a starch-iodide indicator method. An indicator solution was prepared by mixing equal volumes of 20% potassium iodide (KI) and 1% *w*/*v* starch in phosphate buffer (pH 7). To this, 3 mL of diluted ADA solution (1 mL ADA in 250 mL DI water) was mixed with 1.5 mL of the indicator solution. The absorbance of the resulting tri-iodine–starch complex was measured at 486 nm using a UV–Vis spectrophotometer (Model 117, Gujarat, India), as outlined in the methodology reported by Bushra Begum et al. [26].

### 4.4. Preparation of Injectable Hydrogel

The injectable hydrogel was prepared stepwise to ensure homogeneity and reproducibility. First, 7% (*w*/*v*) of aldehyde-modified alginate (ADA, with 70% degree of oxidation) was dissolved in 0.1 M borax under magnetic stirring until a clear solution was obtained. In a separate container, 9% (*w*/*v*) gelatine was dissolved in deionized water at room temperature, using a magnetic stirrer set at 300 rpm until completely solubilized. The gelatine solution was then gradually added to the ADA solution under continuous stirring to promote uniform mixing. Subsequently, 1% (*w*/*v*) polyethylene glycol (PEG) was added to the mixture. The final solution was stirred again to ensure a homogeneous blend. The methodology followed in this study was based on the protocol previously reported by Bushra Begum et al. [26].

### 4.5. Gelation Temperature, Gelling Time, and Rheological Measurements

The gelation temperature and gelling time of the 7% ADA (70%) + 0.1 M borax + 9% gelatin + 1% PEG hydrogel were evaluated at 37 °C using a 10 mL beaker with magnetic stirring. Gelling time was noted when stirring ceased. Rheological properties were assessed in triplicate using a Modular Compact Rheometer MCR 102 (Anton Paar India Pvt. Ltd., Gurugram, Haryana, India) with parallel-plate geometry and Rheoplus/32 software (v3.612006273-33024). Time sweep rheology was performed at 37 °C by rapidly mixing 1 mL of the formulation and placing it on the lower plate. The experiment was carried out at 1 rad/s angular frequency and 10% strain to determine gelation behaviour. The procedure adopted in this study aligns with the methodology described by Bushra Begum et al. [26].

### 4.6. Degradation

The in vitro degradation of the dual-crosslinked hydrogel formulation (7% ADA with 70% oxidation + 0.1 M borax + 9% gelatin + 1% PEG) was monitored in phosphate-buffered saline (PBS) at pH 6.5 for 60 days at 37 °C with weekly buffer changes. Initially, 0.3 g of each hydrogel sample (*W*_0_) was incubated in 10 mL PBS. At predetermined intervals, samples were removed, blotted to eliminate surface moisture, and weighed (*Wt*) [26]. The percentage of weight loss (Δ*W*%) was calculated using the formula:

$$\Delta W\%=\frac{W_{0}-Wt}{W_{0}}\times 100.$$

The detailed other relevant characterization of the hydrogel (7% ADA (70%) + 0.1 M borax + 9% gelatin + 1% PEG), have been thoroughly addressed in our previously published article *Bushra Begum et al*. [26].

### 4.7. Preparation of ADA Hydrogel by Incorporating Rh-TGF-β (Recombinant Transforming Growth Factor-β) for In Vitro Release

As previously described in the procedure outlined by Bushra Begum et al. [26], in this regard, recombinant transforming growth factor-β (Rh-TGF-β) of 1500 ng/30 µL was prepared in tris buffer, homogeneously mixed into the 1 mL of hydrogel precursor solution, and added (400 µL) to a 48-well plate (n = 3), resulting in each well containing 400 μL of hydrogel incorporating 500 ng of Rh-TGF-β. Thereafter, 1 mL PBS (pH = 6.5) was added to each well, and the plates were placed in a 37 °C incubator. At predetermined time points (0, 5, 10, 15, 20, 25, 30, 45, 55, and 60 days), Rh-TGF-β was collected by removing the supernatant and replacing it with an equal volume of PBS. Thereafter, the concentration of Rh-TGF-β was measured by enzyme-linked immunosorbent assay (ELISA). The Rh-TGF-β release experiment was carried out at 37 °C; this is because the temperature of the animals used in subsequent experiments was 37 °C [34]. The amount of Rh-TGF-β released was calculated using the *initial and final concentrations*:

$$\mathrm{percentage}\;\mathrm{of}\;\text{Rh-TGF-β}\;\mathrm{released}=\frac{\mathrm{final}\;\mathrm{concentration}}{\mathrm{initial}\;\mathrm{concentration}}\times 100\;(n=3).$$

### 4.8. Preparation of ADA Hydrogel by Incorporating EGCG (Epigallocatechin-3-Gallate) Injection for In Vitro Release

A total of 50 µM EGCG in 1 mL NaCl solution (0.9%) was homogeneously mixed into the 1 mL hydrogel precursor solution. The EGCG-loaded hydrogel and 3 mL of PBS (pH = 6.5) were added to a 24-well plate and incubated in a shaking bath (37 °C, 50 rpm). From days 1 to 10, the released medium was collected for analysis and replaced immediately with fresh PBS. The EGCG content in the release medium was determined by UV–Vis spectrophotometry at 275 nm (Infinite M200, Tecan, Grödig, Austria) [35].

### 4.9. In Vivo Study

#### 4.9.1. Disc Degeneration Model

**Figure 7:**
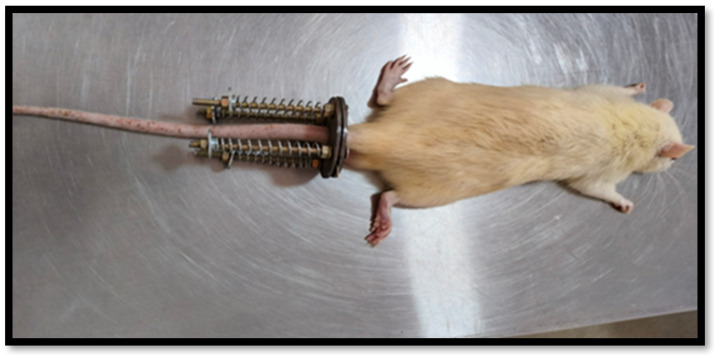
The rat tail is instrumented with an Ilizarov-type apparatus configured for loading the tail. The apparatus consists of aluminum rings, stainless steel threaded rods, calibrated springs, and locking nuts.

In this work, rat tail IVDs were used to induce disc degeneration. As described, similar to that of Iatridis et al., [36,37] animals were anesthetized using intraperitoneal injections of ketamine at a dose of 50 mg/kg, with additional doses administered as needed to maintain anesthesia throughout the procedure. Under anesthesia, an Elizarov-type miniature external fixator was applied and secured at the base of the rat’s tail to serve as the loading apparatus. The device consisted of four threaded rods equipped with springs and two double rings, forming a compact external fixator. Each ring measured 25 mm in diameter and weighed approximately 1 g. The fixator included four small holes (3 mm in diameter) and a central hole (13 mm in diameter), where M2 nuts were used to secure the 2 mm connecting rods. Under anesthesia, the 7th vertebra at the base of the rat’s tail was palpated and marked. A thin, sterile stainless steel wire (0.8 mm in diameter) was then inserted. Using a precision power drill, a similarly sized sterile stainless steel pin was inserted transversely through the 7th vertebral body at a 90° angle—designated as the compression group (Figure 7). Each set of wires was aligned between the two rings, and the vertebral bodies (7th caudal disc) were fixed to the double rings. The rings were then fastened to the connecting rods using nuts. To apply mechanical load, four calibrated springs (with a stiffness of 0.50 N/mm) were mounted onto each threaded rod and tightened against the distal ring. This setup exerted a chronic compressive force on the disc, immobilizing the region and generating a peak compressive stress of approximately 1.3 MPa—equivalent to the pressure exerted on a human lumbar disc during activities like standing up or lifting a moderate weight. Despite the loading apparatus being in place, the rats were allowed free movement. The compressive loading was maintained for 14 days [36,37]. The injectable treatment was started without loading. The hydrogel was injected directly into the centre of the intervertebral disc space (specifically, into the NP region) using a 26-gauge needle. The hydrogel used in this study had a gelation time of approximately 2.5 min, allowing adequate time for injection before in situ gel formation. Post-injection, the localization of the hydrogel within the disc was confirmed during dissection at the experimental endpoint, where the material was observed to be retained within the disc structure, as shown in Figure 3 of the post-compression group. Subgroups of animals were identified with injectable treatment, as shown in Table 1. During surgery and experimental loadings, rats were allowed unlimited food and water, and the instrumented motion segment was allowed to move freely. The effect of these loading and treating conditions on caudal disc properties was assessed by radiological analysis.

#### 4.9.2. Radiological Analysis

Radiographs were obtained two weeks after unloading and twelve weeks following the treatment injection. Throughout the imaging process, meticulous attention was given to maintaining a consistent level of anesthesia to ensure uniform muscle relaxation across all animals, as variations could influence disc height measurements. To ensure accurate comparisons, the preoperative radiograph was consistently used as the baseline reference. All radiographs were digitally scanned and archived using image-capture software version 1.53a [38].

#### 4.9.3. Tissue Processing

After carefully removing the skin, the IVD was excised by making precise cuts through the adjacent vertebral levels. The extracted tissue was then fixed in 4% formaldehyde (Sigma) to preserve its structural integrity. Following fixation, the sample underwent decalcification in a 10% ethylenediaminetetraacetic acid (EDTA) solution to soften the bony components, facilitating further processing. Once the decalcification process was complete and the vertebrae had sufficiently softened, the pins used for stabilization were carefully removed. Finally, the tissue was embedded in paraffin to allow for sectioning and histological analysis [39].

#### 4.9.4. Histologic Preparation

To prepare the tissue for histological analysis, sections of the IVD were carefully stained using two different staining techniques to highlight distinct structural and biochemical features. First, conventional histological staining was performed using hematoxylin and eosin (H&E), which allowed for the visualization of general cellular and tissue morphology. These stained sections were then examined under an upright optical microscope to assess the structural integrity and organization of the disc components. Additionally, Safranin-O staining was employed to evaluate the presence and distribution of proteoglycans within the disc matrix. This specific stain binds to glycosaminoglycans, providing a clear indication of proteoglycan content through variations in colour intensity. The differential staining techniques enabled a comprehensive histological assessment of the disc tissue, aiding in the detailed evaluation of its cellular and ECM composition [39,40].

#### 4.9.5. Immunohistochemistry (IHC)

The paraffin-embedded tissue samples were sectioned into slices of 4 mm thickness for IHC analysis. To prepare the sections for staining, they were first deparaffinized by immersing them in three consecutive changes in xylene, each for 5 min. This was followed by a graded rehydration process, where the sections were sequentially immersed in three changes of 100% alcohol for 8 min each, then in 90% and 80% alcohol for 6–8 min each. The sections were subsequently rinsed thoroughly in distilled water. To remove any residual buffer components, the sections were washed three times with phosphate-buffered saline (PBS). Endogenous peroxidase activity was then blocked by incubating the sections in a 3% hydrogen peroxide (H_2_O_2_) solution for 20 min at room temperature. Antigen retrieval was performed by subjecting the sections to pressure cooking in a 10 mol/L Tris-EDTA buffer (pH 9.0) to enhance antigen accessibility. Following antigen retrieval, the sections were blocked with 0.25% bovine serum albumin (BSA) for 30 min at room temperature to minimize non-specific binding. After removing excess BSA, the sections were incubated with the primary antibody (HSP-90 from Cell Signalling) at an appropriate dilution, either for 1 h at room temperature or overnight at 4 °C. Post-incubation, the sections were rinsed with PBS for two cycles of 4 min each. Next, the sections were covered with an appropriate secondary antibody for 30 min, followed by another washing step using PBS for two cycles of 4 min each. The slides were then treated with a ready-to-use DAB (3,3-diaminobenzidine) substrate buffer along with DAB chromogen to visualize antibody binding. After staining, the sections were washed thoroughly under running tap water. For counterstaining, the sections were immersed in hematoxylin for 3 min, followed by a wash with tap water. Differentiation was performed using 1% acid alcohol. Finally, the sections were dehydrated through a graded series of alcohol concentrations (80%, 90%, and 100%), cleared in three changes in xylene, and mounted using DPX for long-term preservation and microscopic analysis [33,41].

#### 4.9.6. Western Blotting

Approximately 10 mg of rat tail tissue was weighed and homogenized using a pestle and mortar in 100 µL of RIPA lysis buffer (150 mM NaCl, 1.0% IGEPAL CA-630, 0.5% sodium deoxycholate, 0.1% SDS (sodium dodecyl sulphate), and 50 mM Tris, pH 8.0). While careful dissection was performed to isolate the IVD region, the collected tissue specifically included both the NP and AF to represent the major structural components of the disc. The lysate was centrifuged at 21,000× *g* for 20 min at 4 °C, and the clear supernatant was collected for protein quantification (Madhunapantula et al., [42]. The total protein content was estimated using the bicinchoninic acid (BCA) method. A standard curve was prepared using a Pierce™ BCA Protein Assay Kit (Thermo Fisher Scientific, Rockford, IL, USA), with bovine serum albumin (BSA) as the standard. A range of BSA concentrations (25, 250, 500, 750, 1000, 1500, and 2000 µg/mL) was incubated with 200 µL of BCA reagent at 37 °C for 30 min. The BCA reagent consisted of 50 parts reagent A (0.8% sodium bicarbonate, 4% bicinchoninic acid, 0.16% sodium tartrate in 0.1 M sodium hydroxide) and 1 part reagent B (4% cupric sulphate). Absorbance was measured at 562 nm using a multimode plate reader (Thermo Scientific Multiskan GO, Waltham, MA, USA). Appropriately diluted test samples were analyzed similarly, and the protein concentration was determined using the calibration curve. SDS-PAGE gels were prepared using a Bio-Rad mini vertical electrophoresis system (Bio-Rad, Hercules, CA, USA) as per Laemmli’s method. The percentage of gel was selected based on the molecular weight of the target protein. A 5× sample loading buffer (250 mM Tris-HCl, pH 7.0, 50% glycerol, 9% SDS, 0.03% bromophenol blue, and 5% β-mercaptoethanol) was mixed with quantified lysate, heated at 90 °C for 5 min, and centrifuged at 1100× *g* for 30 s. Approximately 100 µg of protein was loaded per well, and electrophoresis was conducted at 100 V for 2–3 h in running buffer (12.4 mM Tris-HCl, pH 8.0, 192 mM glycine, and 0.1% SDS). Proteins were transferred onto a PVDF membrane (Invitrogen, Carlsbad, CA, USA) using pre-chilled transfer buffer (25 mM Tris base, pH 8.0, 190 mM glycine, and 20% methanol) at 25 V overnight. The membrane was blocked with 5% skimmed milk in 10 mM Tris base (pH 7.5) containing 100 mM NaCl for 1 h, rinsed with TBST, and incubated overnight at 4 °C with an HSP90α primary antibody (ABclonal, Cat no-A5006, 1:1000 dilution). After washing, the membrane was incubated for 1 h at room temperature with an HRP-conjugated anti-rabbit IgG secondary antibody (1:5000 dilution) with continuous shaking. Following the transfer, the gel was stained with 0.1% Coomassie Brilliant Blue R-250 in 40% ethanol and 10% acetic acid for 1 h. The staining solution was replaced with a destaining solution (10% acetic acid, 40% methanol, and 2% glycerol) and incubated at room temperature, with changes every 30–60 min until clear protein bands were visible against a sufficiently clear background [23,42].

#### 4.9.7. Statistical Analysis

The results are presented as the mean ± standard deviation, and each experimental sample was replicated at least three times. All statistical analyses were conducted using one-way ANOVA using JMP version 17 software. All showed a confidence level of satisfactory significant difference (*p* < 0.05).

## Figures and Tables

**Table 1:** Summary of Study Design.

Group	Loading Protocol		Vertebral Body (Caudal Vertebrae)	No. of Animals
**Control (Group-1)**	Normal condition		Healthy	3
**Static Load Compression (Group-2)**	Instrumented with rings, rods, and calibrated springs to immobilize and apply axial forces to the instrumented discs		C7	21
	Post compression			3
**Injectable Treated Subgroups unloaded**	**Subgroups**	**Dose**		**A total of 18 animals were selected from the group of 21.**
	(1) Hydrogel	15 µL hydrogel	C7	6
	(2) Hydrogel + Recombinant TGF-β	15 µL hydrogel + 500 ng Rh TGF-β		6
	(3) Hydrogel + Recombinant TGF-β + EGCG	15 µL hydrogel + 500 ng Rh TGF-β + 50 µM EGCG		6

## Data Availability

The original contributions presented in this study are included in the article. Further inquiries can be directed to the corresponding author.

## Abbreviations

The following abbreviations are used in this manuscript:

ADA	Alginate Di-Aldehyde
AF	Annulus Fibrosis
ANOVA	Analysis of Variance
BCA	Bicinchoninic Acid
BMPs	Bone Morphogenetic Proteins
BSA	Bovine Serum Albumin
C7	Caudal disc-7 vertebra
CEP	Cartilage Endplate
DAB	3,3-Diaminobenzidine
DI	Deionized
DPX	Digital Picture Exchange
ECL	Enhanced Chemiluminescence
ECM	Extracellular Matrix
EDTA	Ethylenediaminetetraacetic Acid
EGCG	Epigallocatechin Gallate
ELISA	Enzyme-Linked Immunosorbent Assay
GF	Growth Factor
H&E	Haematoxylin and Eosin
H_2_O_2_	Hydrogen Peroxide
HSPs	Heat Shock Proteins
IDD	Intervertebral Disc Degeneration
IgG	Immunoglobulin G
IHC	Immunohistochemistry
IVD	Intervertebral Disc
IVDD	Intervertebral Disc Degeneration
L	Litre
M	Molar
ml	Millilitre
mm	Millimetre
mM	Millimolar
MW	Molecular Weight
nm	Nanometre
NP and NPCs	Nucleus Pulpous and Nucleus Pulpous cells
NP–EP junction	Nucleus Pulpous–End plate Junction
PBS	Phosphate-buffered saline
PEG	Polyethylene Glycol
PVDF	Polyvinylidene Fluoride
Rh-TGF-β	Recombinant Transforming Growth Factor-Beta
RIPA	Radio-Immunoprecipitation Assay
RPM	Rotation Per Minute
SDS	Sodium Dodecyl Sulphate
SDS PAGE gel	sodium dodecyl sulphate
TBST	Tris-buffered saline with Tween 20
TGF-β	Transforming Growth Factor-Β
U	Numerical units refer to the grayscale intensity values obtained from the radiographic images, which are measured using image analysis software version 1.53a. These values represent the relative radio density of the intervertebral disc region.
UV	Ultraviolet
WB	Western Blot
